# Trade related infections: farther, faster, quieter

**DOI:** 10.1186/1744-8603-1-3

**Published:** 2005-04-22

**Authors:** Ann Marie Kimball, Yuzo Arima, Jill R Hodges

**Affiliations:** 1 Epidemiology, University of Washington, Seattle, USA; 2 Health Services, University of Washington, Seattle, USA

## Abstract

Modern global trading traffics large volumes of diverse products rapidly to a broad geographic area of the world. When emergent infections enter this system in traded products their transmission is amplified. With truly novel emergent infections with long incubation periods, such as Human Immunodeficiency Virus (HIV) or variant Creutzfeld Jacob Disease (vCJD), this transmission may silently disseminate infection to far distant populations prior to detection. We describe the chronology of two such "stealth infections," vCJD and HIV, and the production, processing, and distribution changes that coincided with their emergence. The concept of "vector products" is introduced. A brief case study of HIV incursion in Japan is presented in illustration. Careful "multisectoral" analysis of such events can suggest ecologically critical pathways of emergence for further research. Such analyses emphasize the urgency of implementing safety measures when pathogens enter globally traded products.

## Review

### Introduction

The global trading system today speeds unprecedented volumes of product to unparalleled numbers of markets throughout the world. Coincident with its growth has been the unwelcomed emergence and dissemination of new infections in human populations. This article will examine linkages between these two phenomena through the optic of "stealth" infections – emergent infections with long, silent incubation periods. When such pathogens are carried in a globally traded product (*vector product*), spectacular geographic amplification can occur prior to the onset of clinically recognizable illness. Examples of these trade-related emerging infections caused by vector products include variant Cruetzfeldt Jacob disease (vCJD) and HIV/AIDS. Vector products can be defined as those products that carry pathogens. It should be noted, however, that "vector" in infectious disease parlance usually refers to living organisms, such as mosquitoes. The use of the term here with regards to product is expedient, albeit non-conforming to normative use.

The drivers for broad marketing of the "vector products" in these two pandemics were 1) promotion and growth of global demand for beef and animal feed and 2) promotion and growth of global demand for human anticoagulant factors for medical treatment. These market drivers provoked a marked "ramping up" of production in pace and capacity for the vector product. The production of these vector products involved pooling of large amounts of biological material. In each case novel pathogens emerged and were disseminated over a broad geographic area prior to their clinical detection.

### Mad Cow Disease and vCJD

Global trade trends for the products implicated in the transmission of HIV and vCJD reflect similar scales of increase in volume and dollar amounts during the decade prior to the detection of human diseases.

The incubation period of vCJD is variable, estimated at up to 15–30 years [1]. The incubation period of BSE (Bovine Spongiform Encephalopathy), or mad cow disease, in cattle is also on the order of years, which has complicated culling interventions [2]. Mad cow disease was first detected in British herds in 1986. Wide-scale trade embargoes did not occur until 2000, although some products, such as bull semen, were embargoed as early as 1996, when BSE was first linked to human vCJD [3].

**Figure 1:**
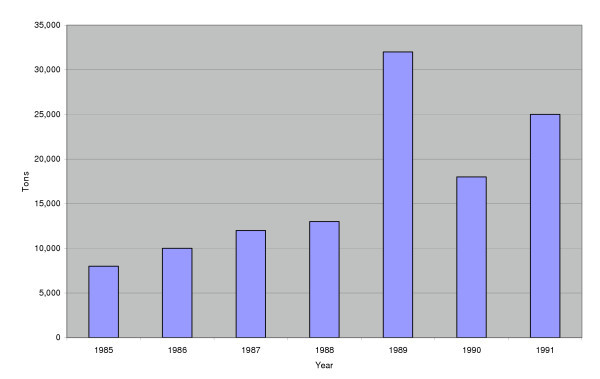
UK Exports of Meat-and-Bone Meal, in tonnes, 1985 – 1991*. * legal for swine and poultry feed at the time; above scenario also replicated in other countries. Source: Butler, 1996[4].

In the case of beef and bone meal, the suspected source of BSE, exports of the implicated product doubled in 1989, from approximately 15,000 tons in 1988 to more than 30,000 tons (Figure 1) [4]. The surge in exports was at least partly due to precautions taken as a result of concerns about potential disease risk. Meat and bone meal (MBM), a side product of animal slaughter, has long been appreciated as a source of protein for animal feed. In fact, it is speculated that BSE was introduced in cattle when scrapie-infected material from sheep was pulverized into cattle feed years ago [5]. Thus when BSE surfaced in the mid-1980s, the UK banned the use of ruminant-derived meat and bone meal in ruminant diets in 1988. However, the feed was still permitted for non-ruminants. The ban caused the price of the British MBM feed to drop, making it more attractive for international trade. Once the feedstuffs moved through Europe, they were used for a variety of purposes, including feed for ruminants; unlike in the UK, in continental Europe, bone meal was a legal commodity for all animal feeds.

**Figure 2:**
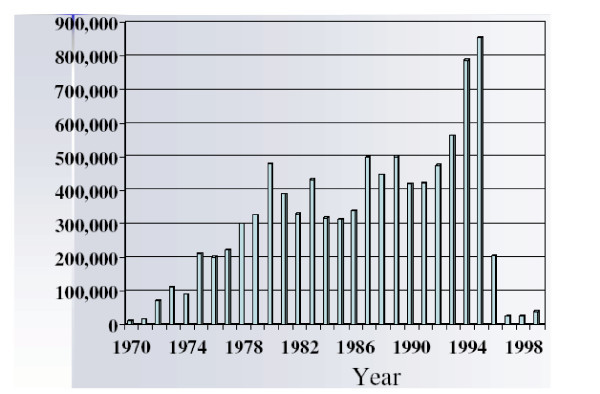
UK Beef Exports, in 1,000 U.S. Dollars, 1970 – 1999. Source: Food and Agriculture Organization of the United Nations [6]

Meanwhile, the value of UK beef exports more than doubled between 1985 and 1995 (Figure 2) [6]. A superficial glance at the data would suggest a ramping up of production; however, the UK beef industry, like most industries, is more complex. In fact, as of 1995, the production of beef in the UK had been static for two decades at about one million tons annually [7]. But consumption of beef in the domestic market was declining, partly associated with changing lifestyles. In 1980 Britons consumed 21.3 kg per capita; in 1993 this fell to 17.0 kg per capita. In response, consumer pricing for beef declined, while production and distribution costs remained largely static. Consequently, trade had, by 1995, become an important factor, representing 21 to 23 percent of the total market [7].

Prices fell both in response to declining domestic consumption and the implementation of the General Agreement on Tariffs and Trade (GATT) reforms, which proposed liberalization of agricultural trade through reduced border protection, domestic support payments and export subsidization [7]. The GATT agreements committed economies to reduce tariffs on imports, and in effect, there was price pressure to be competitive to keep their market share at home, to become more efficient. With margins in the supplying industries squeezed [7], there was market pressure for consolidation of the previously fragmented production system. Farms of 50 cows or more increased from 6,400 to 9,000 between 1986 and 1991.

These changes in processing may have permitted prions to cross the species barrier from ruminant to human. Rendering had been a "batch" procedure in Europe and North America, relying on very high solvent extraction for the production of tallow. In the 1970's, a continuous system of rendering was introduced that relied on vacuum at relatively low temperatures and produced high quality tallow. This efficient process saved costs to the industry. After the BSE crisis, EU studies of abbattoir practices suggested that the processes in place in the United Kingdom in the 1990's were ineffective in deactivating prions [8].

### Amplification and Transmission through Trade

The geographic distribution of BSE is broad. If in fact the emergence of this agent occurred only in Britain and bone meal and animal feed contamination occurred only from this source in the late 1980's it is impressive that OIE (World Organisation for Animal Health) lists only four countries currently confirmed as BSE-free [9]. By 2004, in addition to the UK, 22 countries had notified the OIE of cases of BSE in farmed cattle. Cases have been reported largely in Europe but also in North America and Asia [9]. The UK may not in fact have been the sole origin, given a recent report which suggests France had undetected BSE epidemics on a large scale during the same time period [10].

The BSE/vCJD emergence has also impacted the human blood supply. Based on the experience with HIV/AIDS, blood banks are concerned that an agent that cannot be screened for may enter the global blood supply, amplifying infectious transmission. This concern has been heightened by the occurrence of vCJD apparently related to blood transfusion [11]. Thus exclusion criteria in the United States and blood donation fractionation in the UK reflect measures to mitigate this risk.

### HIV/AIDS

By the end of 2003 UNAIDS estimated that almost 40 million people were living with HIV/AIDS [12]. While human sexual contact is the major global route of infection of HIV, the history of the pandemic also documents the wide-ranging infection due to infected blood products. Again, innovations in product in response to a global market were apparently correlated with amplification and dissemination of product. In the early 1980's, when AIDS was described in the first cluster of young gay men in San Francisco, ongoing concern about the risk of hepatitis in pooled blood for manufacture of blood factors had spurred producers to a search for a means of screening their product for hepatitis virus. However, viral deactivation processes were not generalized in the industry when HIV emerged; with a profitable international market, the new lyophilized blood product was globally traded.

The commercial sector for the production of blood factors is most developed in the United States. Data from the U.S. Census Bureau attests to a growing trade in blood product from the mid-1970s to the late 1980's, from approximately 50 million USD in 1975 to 325 million USD in 1988. We now know that half of the hemophiliacs in the U.S. were infected with HIV through this route, and an untold number of hemophiliacs worldwide. In some countries, such as Japan, this was probably the primary route of entry into the population. By the end of the 1990's, there were 400 commercial centers for plasmapheresis operating in the U.S. These centers, which employ paid donors, provided 60% of the worldwide requirement for plasma [13].

By mid 1982 the possible link between AIDS and the blood supply was reported in the CDC's Morbidity and Mortality Weekly Report, and was widely known and accepted the following year with the occurrence of cases in hemophiliacs living in geographically dispersed areas. But retooling the fast-growing industry of factor production posed substantial difficulties, for several reasons. 1) The science of HIV/AIDS disease was still early in its evolution, and the data were not therefore clearcut 2) the plasmapheresis industry utilized largely compensated donors (who were often higher risk for HIV/AIDS) as a basis for obtaining plasma, 3) the most heavily impacted group, homosexual males, were wary of discrimination in measures adopted. The initial self-exclusion strategies, for example, did not ask donors about homosexual sexual practice for fear of discrimination.

In early 1983 both the voluntary and commercial sectors had taken some measures for reducing the participation of high risk donors in plasmapheresis. But the Inquiry on the Blood System in Canada, published by Health Canada's Krever Commission in 1997, notes, "There is evidence, however, that the voluntary sector refused to stop collecting in high risk areas, though its blood donor recruiting officials no longer targeted high risk individuals, and that the commercial sector also continued to operate in such areas" [13]. Viral inactivation methods had been in development since the early 1970's to try to cut down on hepatitis transmission in blood. However, the industry leaders considered such steps proprietary information for production and so the work towards successful strategies was not shared across the corporate competitors. In 1984 the major producers had all been licensed to distribute heat-treated products to cut down on the threat of hepatitis and AIDS infection.

As with any product, blood products can be subject to a recall, which is initiated when the public's health is at risk due to contamination of the product. This option was available to the US Food and Drug Administration as soon as the risk of HIV transmission in blood factors became known in March of 1983. An Institute of Medicine study convened years later to review the history was critical of the absence of a cogent, strong recall policy [14].

By 1985 the ELISA screening test for HIV came into use. This, coupled with heat treatment of the factor product, has markedly reduced the risk of HIV transmission in blood. However, global spread of the virus, in part facilitated by the global trade in factor VIII, had already occurred in the 1980's. This contributed to the profound global reach of the epidemic seen today. The experience of Japan illustrates this point.

### Japan, Factor VIII and HIV/AIDS

In 1994, the Tenth International AIDS Conference was held in Japan, focusing on subgroups, such as hemophiliacs, that had previously received less attention [15]. Although Japan had been characterized by some as reluctant to acknowledge the introduction of HIV to Japan via "sexual tourism," the virus did initially hit Japan through the blood supply. According to the World Federation of Hemophilia, the majority of Japanese hemophiliacs are thought to have been affected during 1983–1985 by non-heat-treated factor concentrates imported from the US. In fact, by 1985 the US provided 90 percent of imported factor concentrates in Japan [13], and the Japanese AIDS Research Group reported that the causative agent of AIDS was found in factor VIII concentrates [13]. Of 5,000 hemophiliacs in Japan, approximately 2,000 had been infected with HIV/AIDS by 1997 according to the Krever Report. The occurrence of HIV/AIDS through factor concentrates became known in Japan as "Yakugai" AIDS (meaning "AIDS due to the harmful effect of medicine").

As factor concentrates were manufactured by pooling plasma from many donors, the entire pool could be contaminated by a single unit from an HIV-infected donor. Dr. Michael Rodell, former vice-president of the Armour Pharmaceutical Company, estimated that four infected persons could contaminate the entire world supply of factor VIII [13].

The Ministry of Health and Welfare in Japan regulates the national blood system by licensing the use of blood products. In 1983 the Japanese AIDS Research Group, the central technical group advising the Ministry on prevention and control policy, reportedly considered a proposal from the US fractionator Travenol to allow the importation of heat-treated (virus-inactivated) factor VIII. At that time, a new product could receive regulatory approval without undergoing clinical trials if it could be classified as a "change to manufacturing method having no effect on the effective ingredients" [13].

The Ministry rejected the idea, apparently mistrusting the reliability of the US FDA tests and fearing side-effects from heat-treated concentrates [13]. Thus, while US reports in 1983 showed that HIV could be contracted from contaminated factor concentrates, the Ministry continued the import of non-heat-treated factor concentrates, with the stipulation that they be accompanied by a certificate that they did not contain plasma from donors at high risk of contracting HIV/AIDS [13]. The use of imported non-heat-treated concentrates increased in 1984 and peaked in 1985 [13]. Although heat-treated factor concentrates were approved for use in 1985 – because the Ministry recommended that physicians continue to prescribe non-heat-treated factor concentrates (and blood product manufacturers continued to distribute them without warning labels about the risk of AIDS) – many hemophiliacs continued to use non-heat-treated concentrates through 1986 [13]. A further exacerbating problem was the fact that some health care providers neglected to inform their hemophiliac patients of their HIV infection, allowing their sexual partners to become infected [16].

This tragedy in Japan was due to a combination of factors in government, industries, and medical communities [17]. The former chief of the Office of Biologics at the Ministry of Health and Welfare, the head of the AIDS Research Group, and three Green Cross executives were indicted for misconduct and/or professional negligence. Still, the "Yakugai AIDS" scandal is not over, and trials continued through 2004.

## Conclusion

The following similarities are thus apparent for infections with very long incubation periods in the modern era of trade and travel: 1) global forces such as market demand or GATT provisions that favor increased exports also favor consolidation, which may set the stage for streamlining processing of product 2) such streamlining of production for efficiency and cost savings may have a role in emergence of new infections when biological materials are the basis for product formulation 3) the emergence of new pathogens that are disseminated through trade and travel creates a "science gap" where rapid research is imperative to find and implement agent-specific safety strategies before extensive infection occurs 4) once clinical disease is manifest widespread dissemination of infection has occurred and risk can be mapped using product specific trade data. This mapping may allow timely institution of surveillance.

Among the most sobering lessons from these two emergent diseases are the consequences of the division between public and private science and the division between the world of health and the world of trade. When heat treatment processes were developed for factor VIII, they were not shared across the industry in an expeditious fashion. Urgent information on product risk and on safety innovations is crucial to public safety and timely public health intervention in disease.

The "sectors" of global trade and global health have few working links. Information flow between the international agencies, the World Trade Organization and World Health Organization, while improving, is not routine. At the regional and national level, intersectoral communications between the trade and health communities is also troublesome. The European Union stands in contrast to this generalization, with extensive collaboration in disease surveillance among its members [18]. The regional initiatives on emerging infections of such regional trading groups as the Asia Pacific Economic Cooperation (APEC), ASEAN and others are encouraging. At the national level, formal collaboration between ministries of commerce and health on product investigation or tracing is sparse.

British authorities were prescient in their implementation of the "Offal Ban" in 1988 [19]. While the risk of vCJD from BSE food animals was not established, they took precautionary measures to limit exposure which were, in retrospect, prudent. At the same time, the issue of potential "opportunistic exporting" of animal feed by the UK onto the international market, in response to the bans imposed domestically on such feed, has been raised. If precautionary recall of factor VIII had been fully implemented in 1986, it is clear that hundreds of cases of HIV may have been averted, and the geographic extent of human infection limited. Currently the costs and effectiveness of the "precautionary principle" in commerce are under discussion [20]. Accurate and complete description of these two global emergent diseases (through mathematical modeling and other research) will be important in informing those discussions.

In summary, two novel infections with long incubation periods, HIV/AIDS and BSE/vCJD, are illustrative in describing the wide geographic dissemination and urgent consequences of emergent infections associated with globalized trade.

## Competing interests

The author(s) declare that they have no competing interests.

## Authors' contributions

Ann Marie Kimball and Yuzo Arima drafted and edited the manuscript. Jill Hodges reviewed and edited the manuscript.
